# Association between Cystatin C and MRI Measures of Left Ventricular Structure and Function: Multi-Ethnic Study of Atherosclerosis

**DOI:** 10.4061/2011/153868

**Published:** 2011-10-01

**Authors:** Subhashish Agarwal, Vinay Thohan, Michael G. Shlipak, Joao Lima, David A. Bluemke, David Siscovick, Antoinette Gomes, David M. Herrington

**Affiliations:** ^1^Department of Cardiology, Wake Forest University, Winston-Salem, NC 27157, USA; ^2^University of California, San Francisco, CA 94143, USA; ^3^Division of Cardiology, John Hopkins University, Baltimore, MD 410-516-8000, USA; ^4^Division of Cardiology, John Hopkins University and Imaging Sciences Training Program, National Institutes of Health and National Institute of Biomedical Imaging and Bioengineering, Bethesda, MD 20892, USA; ^5^Cardiovascular Health Research Unit, University of Washington, Seattle, WA 98195-5502, USA; ^6^UCLA David Geffen School of Medicine, CA 90095, USA

## Abstract

*Introduction.* Reduced kidney function, approximated by elevated cystatin C, is associated with diastolic dysfunction, heart failure, and cardiovascular mortality; however, the precise mechanism(s) that account for these relationships remains unclear. Understanding the relationship between cystatin C and subclinical left ventricular (LV) remodeling, across ethnically diverse populations, may help explain the mechanisms underlying the association of kidney dysfunction with heart failure and cardiovascular mortality. *Methods.* Measures of cystatin C and LV parameters were obtained from the multi-ethnic study of atherosclerosis (MESA) cohort at baseline (*N* = 4, 970 with complete data on cystatin C and LV parameters). LV parameters; LV end-diastolic (LVEDV) and end-systolic volumes (LVESV), LV mass (LVM), concentricity (LV mass/LV end-diastolic volume), and LV ejection fraction (LVEF) were measured using magnetic resonance imaging. Nested linear models were used to examine the relationship between higher quartiles of cystatin C and LV parameters, with and without adjustment for demographics, height, and weight, and traditional cardiovascular risk factors. Similar analyses were performed stratified by ethnicity and gender. *Results.* A fully adjusted model demonstrated a linear relationship between higher quartiles of cystatin C and lower LVEDV, (Mean ± SE, 128 ± 0.7, 128 ± 0.7, 126 ± 0.7, 124 ± 0.8 mL; *P* = 0.0001). Associations were also observed between higher quartiles of cystatin C and lower LVESV (*P* = 0.04) and concentricity (*P* = 0.0001). In contrast, no association was detected between cystatin C and LVM or LVEF. In analyses stratified by race and gender, the patterns of association between cystatin C quartiles and LV parameters were qualitatively similar to the overall association. *Conclusion.* Cystatin C levels were inversely associated with LVEDV and LVESV with a disproportionate decrease in LVEDV compared to LVM in a multi-ethnic population. This morphometric pattern of concentric left ventricular remodeling, may in part explain the process by which kidney dysfunction leads to diastolic dysfunction, heart failure and cardiovascular mortality.

## 1. Introduction

Elevated cystatin C, a marker of reduced kidney function, is associated with increased cardiovascular mortality [1] and incident heart failure [2–4]. Cystatin C has been shown to be a superior marker of renal function compared to creatinine or estimated GFR as it is less affected by age, gender, and ethnicity [5–8]. Moreover, higher levels of cystatin C have been shown to be associated with increased LV mass [9], concentric LV hypertrophy [9, 10], and diastolic dysfunction [11]. An association between cystatin C and heart failure, particularly diastolic heart failure, is not unexpected given that it is a sensitive and specific marker of renal insufficiency and highly correlates with hypertension [9]; however, various lines of evidence suggest that the relationship between cystatin C and LV mass and hypertrophy may go beyond simple confounding due to its association with hypertension [11]. Data are needed to better define the relationship between cystatin C and ventricular remodeling, which may clarify its relationship with heart failure and cardiovascular mortality and elucidate new mechanisms which may lead to development of novel therapies. Further, this will clarify the process by which mild-moderate kidney dysfunction may lead to ventricular remodeling and heart failure. We hypothesize that cystatin C is a marker for ventricular remodeling, independent of blood pressure, in a multi-ethnic population.

## 2. Materials and Methods

### 2.1. Study Population

The Multi-Ethnic Study of Atherosclerosis (MESA) design has been previously described [12]. Briefly, MESA is a prospective cohort study that began in July 2000 to investigate the prevalence, correlates, and progression of subclinical CVD. The study included 6814 racially diverse men and women aged 44–84 years old recruited from 6 US communities (Baltimore, Md; Chicago, Ill; Forsyth County, NC; Los Angeles County, Calif; northern Manhattan, NY; and St. Paul, Minn). MESA cohort participants were 38% Caucasian (*n* = 2,622), 28% African-American (*n* = 1,893), 22% Hispanic (*n* = 1,496), and 12% Chinese (*n* = 803). Individuals with a history of physician-diagnosed myocardial infarction, angina, heart failure, stroke, or transient ischemic attack, or who had undergone an invasive procedure for CVD (coronary artery bypass graft, angioplasty, valve replacement, pacemaker placement, or other vascular surgeries) were excluded from the study at baseline (2000–2002). This study was approved by the Institutional Review Boards of each study site, and written informed consent was obtained from all participants.

### 2.2. Laboratory Measures and Data Collection

Medical history, anthropometric measurements, and laboratory data for the present study were taken from the first examination of the MESA cohort (July 2000–August 2002). Information about age, sex, ethnicity, and medical history was obtained by questionnaires. Resting blood pressure was measured using Dinamap Monitor PRO 100 (Critikon, Tampa, Fl) automated oscillometric device. Three measurements were obtained at 1-minute intervals with the subject in the seated position with back and arm supported after 5 min of rest with an appropriate-sized cuff, with the cuff at the level of the heart, using a standardized protocol. The average of the second and third measurements was recorded as the resting blood pressure. Hypertension was defined as a systolic blood pressure ≥140 mm Hg, a diastolic blood pressure ≥90 mm Hg, or currently taking medications for blood pressure control. Smoking use was defined as never, former, and current smokers. Diabetes was defined as a fasting glucose ≥126 mg/dL or use of insulin or hypoglycemic medications. Total cholesterol was measured from blood samples obtained after a 12-hour fast and measured using a standardized kit (Roche Diagnostics). Cystatin C was measured from frozen sera at a central laboratory (University of Vermont, Colchester, Vt) using a BNII nephelometer (Dade Behring Inc, Deerfield, Ill) and a particle-enhanced immunonephelometric assay (N Latex Cystatin C; Dade-Behring) [13]. The analytical coefficient of variation for this assay is 2.5%.

### 2.3. Magnetic Resonance Imaging Protocol

The MESA MRI protocol has been described in detail elsewhere [14]. Briefly, cardiac MRI testing was performed at MESA study sites using a standard protocol and read at a central site (Johns Hopkins University, Baltimore, MD). LVEDV and LVESV, LVM and LVEF were determined using 1.5-Tesla MR scanners: Signa LX and CVi (GE Medical Systems, Waukesha, Wis) and Symphony and Sonata (Siemens Medical Systems, Erlangen, Germany). MRI was performed with a 4-element phased-array surface coil placed anteriorly and posteriorly, with electrocardiogram gating and brachial artery blood pressure monitoring. Imaging consisted of cine images of the left ventricle with a temporal resolution of 50 milliseconds or less. LVEDV and LVESV were calculated using Simpson's rule (the summation of areas on each separate slice multiplied by the sum of slice thickness and image gap). LVM was determined by the sum of the myocardial area (the difference between endocardial and epicardial contour) times slice thickness plus image gap in the end-diastolic phase multiplied by the specific gravity of myocardium (1.05 g/mL). LVEF was calculated as LV stroke volume divided by LVEDV multiplied by 100. Concentricity was determined by obtaining a ratio of LVM by LVEDV. The interobserver variability in estimating LV parameters was: LVEDV, technical error of the mean (TEM%, 95% CI), (4.4 mL, 95% CI 2.6, 6.6); LVESV (12.8 mL, 95% CI 9.3, 16.2); LVM (6.0 gm, 95% CI, 4.6, 7.4); LVEF (5.1%, 95% CI 3.6, 6.7) and intraobserver variability in estimating LV parameters was: LVEDV (5.1 mL, 95% CI, 3.94, 6.15); LVESV (10.5 mL, 95% CI, 8.17, 12.68); LVM (6.3 gm, 95% CI, 5.17, 7.38); LVEF (3.9%, 95% CI, 3.06, 4.72) [14].

### 2.4. Statistical Methods

Differences in baseline characteristics were compared across quartiles using analysis of variance (ANOVA) for continuous variables and Chi-square tests for categorical variables. The LV parameters of interest, LVEDV, LVESV, LVM, LVEF, and concentricity (LVM/LVEDV), were compared across cystatin C quartiles using ANOVA. 

Multivariate linear regression analyses in nested models were performed to evaluate the association of cystatin C quartiles, with the following outcomes: LVEDV, LVESV, LVM, LVEF, and concentricity (LVM/LVEDV). After univariate analysis (Model 1), the following covariates were considered as potential confounders: age, gender, race/ethnicity, height, and weight (Model 2); Model 2 covariates plus traditional cardiovascular risk factors: history of diabetes, antidiabetic medications, hypertension, systolic blood pressure, total cholesterol, antilipid medications, and smoking (Model 3). Similar linear analyses in nested models were performed stratified by ethnicity and gender. Interaction terms using (cystatin C quartile × race) and (cystatin C quartile × gender) were introduced in the model. 

In a separate analysis, where serum creatinine or creatinine-based estimated glomerular filtration rate (eGFR-CR) was added in the model to assess the association of cystatin C quartiles with LV parameters, collinearity diagnostics such as variance inflation factor was performed to avoid unstable parameter estimates. Sensitivity analysis between log transformed cystatin C and LV parameters was also performed. All statistical analyses were performed using JMP Version 8 (SAS Institute Inc., Cary, North Carolina).

## 3. Results

### 3.1. Baseline Characteristics by Cystatin C Quartiles

Of 6,814 MESA participants, 1,786 did not have MRI measures of LV structure and function, and 58 had no serum cystatin C measures, leaving 4,970 participants who were included at baseline for analysis. Cystatin C levels ranged from 0.36 to 7.59 mg/L with a mean value of 0.89 mg/L and a median of 0.86 mg/L (interquartile range: 0.76 to 0.98 mg/L). For ease of comparability with previous studies, cystatin C was divided into quartiles: quartile 1 (≤0.76 mg/L), quartile 2 (>0.76 and ≤0.86 mg/L), quartile 3 (>0.86 and ≤0.98 mg/L), and quartile 4 (>0.98 mg/L). Participants with higher concentrations of cystatin C were more likely to be older, to have diabetes and hypertension, and to use antihypertensive medications. Those with higher cystatin C levels were less likely to be Chinese or black and had higher body mass indices. As expected, increasing quartiles of cystatin C were associated with higher levels of creatinine and lower creatinine-based estimated glomerular filtration rates (Table 1).

### 3.2. Cystatin C Quartiles and LV Parameters

**Figure 1:**
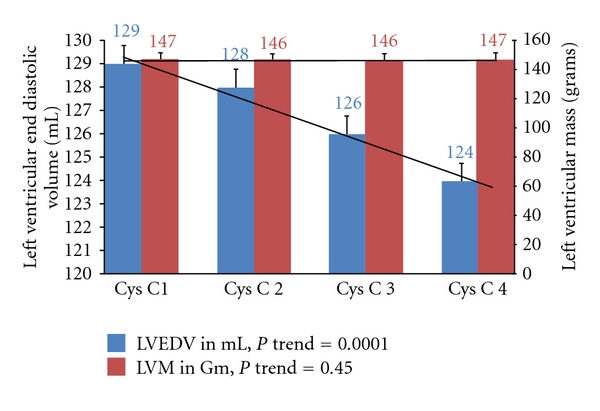
*P* trend in a fully adjusted model between Cystatin C quartiles and LVEDV and LVM in the MESA cohort (2000–2002). Cystatin C Quartiles 1: ≤0.76, Quartile 2: >0.76 & ≤0.86, Quartile 3: >0.86 & ≤0.98, Quartile 4: >0.98; LVEDV, LV End Diastolic Volume; LVM, LV Mass; Model adjusted for age, gender, race/ethnicity, height, weight, diabetes, use of anti-diabetic medications, hypertension history, systolic blood pressure, total cholesterol, anti-lipid medications, and smoking.

The unadjusted associations and *P* values for trend between higher cystatin C quartiles and MRI measures of LV remodeling are demonstrated in Table 2. In a multivariate linear analysis, adjusting for age, gender, race/ethnicity, height, and weight (Model 2), increasing quartiles of cystatin C were significantly associated with LVEDV and concentricity. This association remained after further adjustment for history of diabetes, antidiabetic medications, hypertension, systolic blood pressure, antihypertensive medications, total cholesterol, antilipid medications, and smoking (Model 3) (Figure 1). There was no relationship between cystatin C and LVM or EF in a fully adjusted model (Figure 1). Next, we assessed the influence of race/ethnicity and gender on the associations between cystatin C quartiles and LV parameters in a fully adjusted model. The associations observed between cystatin C and LV parameters were qualitatively similar within race subgroups and in both genders.

 In a separate analysis after further adjustment for serum creatinine, the association between cystatin C quartiles and LV remodeling (LVEDV *P* = 0.0001, LVESV *P* = 0.04, and LVM/LVEDV *P* = 0.0001) remained highly significant. The results of collinearity diagnostics revealed a variance inflation factor (VIF) of 1.14 for both creatinine and cystatin C, with creatinine and cystatin C in the model, and a VIF of 1.37 and 1.47, for creatinine and cystatin C, respectively, in the full model. 

In another separate analysis, after further adjustment for creatinine-estimated GFR (eGFR-CR) instead of creatinine in the full model, associations between cystatin C quartiles and LV parameters: LVEDV (*P* = 0.0004), LVESV (*P* = 0.03), and LVM/LVEDV (*P* = 0.0001) remained highly significant. The results of collinearity diagnostics revealed a variance inflation factor (VIF) of 1.33 for both eGFR-CR and cystatin C, with only eGFR-CR and cystatin C in the model, and a VIF of 1.55 and 1.66, for eGFR-CR and cystatin C, respectively, in the full model. The associations between log transformed cystatin C and LV parameters were similar, and hence data was not presented.

## 4. Discussion

In this large, ethnically diverse population of 4,970 individuals of ages 44–84, free of cardiovascular disease, cystatin C was inversely associated with LV end-diastolic and end-systolic volumes and directly associated with concentricity independent of hypertension, antihypertensive medications, and traditional cardiovascular risk factors. The association observed between cystatin C and LV parameters was not modified by ethnicity or gender. No association was demonstrated between cystatin C and LV end-diastolic mass and LV ejection fraction overall in the population or when stratified by ethnicity and gender. 

Ventricular remodeling is a broad term which refers to deviation of normal cardiac structure with notable changes in chamber volumes, wall thickness, and shape [15]. Ventricular remodeling is influenced by the type and duration of cardiovascular injury, and genetic influences [16]. Ventricular remodeling incorporates various morphometric patterns including concentric LV remodeling (increased ratio of LV mass to end-diastolic volume) [17]. Progressive concentric LV remodeling is a key feature of diastolic dysfunction [18], diastolic heart failure [19], and cardiovascular morbidity and mortality [20, 21]. 

In the Heart and Soul cohort [4, 11] with echocardiographic measures of LV structure in approximately 800 participants with coronary artery disease, cystatin C was associated with LV hypertrophy, diastolic dysfunction, and incident heart failure. However, the relationship between cystatin C and LV volumes was not evaluated. Similarly, Patel et al. [9] in the Dallas Heart Study (DHS) found an association between cystatin C and LV mass, concentric hypertrophy, and LV wall thickness after adjusting for cardiovascular risk factors in a cohort of 2,500 Caucasians and African-Americans. However, the authors did not find significant association between cystatin C and LV volumes, unlike our study, perhaps reflecting a younger cohort age (DHS, 30 to 65 years versus MESA 44 to 84 years) and/or shorter duration of renal dysfunction. An association between mild-moderate kidney dysfunction and LV hypertrophy was demonstrated in 4,971 MESA participants using cystatin C-derived GFR, but the study did not address the association of kidney dysfunction and LV volumes [10]. The current study extends and corroborates previous findings into a larger multisite multi-ethnic population free of cardiovascular disease by exploring the relationship between cystatin C and LV volumes and concentricity with more precise measures of cardiac structure and function using magnetic resonance imaging.

Koenig et al. [22] studied 1,033 subjects and found a stronger association of cystatin C with CVD events compared to creatinine or estimated GFR, suggesting that current estimates of renal function do not fully explain renal dysfunction particularly in mild-to-moderate stages. Similarly, Patel et al. [9] after adjusting for creatinine and GFR in their models demonstrated significant association between cystatin C and concentric hypertrophy. In this study, we adjusted for known risk factors and further adjusted for creatinine or creatinine-estimated GFR and found significant association between cystatin C quartiles and LV parameters, which corroborates with such previous findings [9, 22].

The mechanisms of association between cystatin C and cardiovascular disease are not fully characterized, and several complementary hypotheses can be envisioned. Renal insufficiency is a known risk factor for heart failure [23], diastolic dysfunction, and LV remodeling [24]. The risk of cardiovascular remodeling attributable to mild-to-moderate renal insufficiency may not be fully captured by current estimates of renal function such as creatinine, GFR, and creatinine clearance. Thus, as a more precise marker of renal function [6], cystatin C is associated with ventricular remodeling. Second, cystatin C is highly correlated with hypertension [9], and the observed association between cystatin C and LV remodeling could be the result of adjustment by imprecise measures of blood pressure leading to residual confounding. Finally, cystatin C may play a direct physiologic role and influence ventricular remodeling independent of hypertension and renal function. The balance between cysteine proteases (cathepsins B, S, and K) and cysteine protease inhibitors (cystatin C) has been implicated in pathological LV remodeling in heart failure [25, 26]. Cathepsins, stored in lysosomes, are responsible for the physiological digestive turnover of cellular molecules, and abnormal levels of which may adversely influence cardiac remodeling [26]. Although the mechanism between cystatin C and LV remodeling is unclear, it is possible that the alteration of the balance between these two classes of proteins may contribute to the LV remodeling process by matrix degradation [26]. 

The strength of this study includes a large ethnically diverse population with stringent quality control procedures and MRI measurements of cardiac structure and function. The present study extends prior investigations and supports the view that kidney disease approximated by elevated cystatin C is associated with concentric remodeling. We found modest changes in LV volumes across higher quartiles of cystatin C after adjustment which was found to be statistically significant. The clinical implications of these findings are not yet clear. It remains to be seen whether the pattern of LV remodeling demonstrated in the current study is associated with subsequent development of clinical heart failure or cardiovascular mortality in the MESA cohort. 

We acknowledge several limitations. MESA did not directly measure glomerular filtration rate; therefore, we cannot be certain that the association between elevated cystatin C level and LV parameters are solely caused by its approximation of impaired GFR. Due to the cross-sectional design, the study cannot address the direction of the association between cystatin C and LV remodeling, and the observed associations could be purely a statistical phenomenon; however, the results are consistent with previous such observed relationships which point towards a true association. The association observed between cystatin C and LV remodeling could result from decreased renal perfusion from a reduced cardiac output, or structural remodeling of arteries, although these possibilities were minimized by inclusion of a cardiovascular disease-free population, though self-reported history may be imperfect due to recall bias. Although efforts were made to adjust for known confounders, there remains a possibility of failure to adjust for unknown confounders or inadequate adjustment of established risk factors (severity and duration of hypertension, diabetes) resulting in spurious results due to residual confounding. Some studies suggest that corticosteroids [27] and thyroid function [28] are associated with cystatin C, and since adjustment with these measures was not performed, results should be interpreted with caution in this subset of individuals.

## 5. Conclusion

Cystatin C levels are inversely associated with LV end diastolic and systolic volumes and directly associated with concentricity independent of traditional cardiovascular risk factors including hypertension in a multi-ethnic population. This morphometric pattern of concentric LV remodeling may in part explain previously observed associations between cystatin C and diastolic dysfunction, heart failure, and cardiovascular mortality.

## Figures and Tables

**Table 1:** Baseline characteristics of 4,970 MESA participants by quartile of cystatin C.

Variables	Quartile 1	Quartile 2	Quartile 3	Quartile 4	*P* value
Demographics					

(*n* = 4,970)	(*n* = 1,347)	(*n* = 1,356)	(*n* = 1,150)	(*n* = 1,117)	
Age, in years	56.5 (8.6)	59.7 (9.3)	63.4 (9.3)	67.8 (9.6)	<0.0001
Male	511 (38%)	647 (48%)	621 (54%)	591 (53%)	<0.0001
Female	836 (62%)	709 (52%)	529 (46%)	526 (47%)	<0.0001
Caucasian	453 (33%)	505 (37%)	484 (42%)	501 (45%)	<0.0001
Chinese	234 (17%)	172 (13%)	129 (11%)	116 (10%)	<0.0001
African	384 (29%)	359 (26%)	257 (22%)	274 (25%)	<0.003
Hispanic	276 (20%)	320 (24%)	280 (24%)	226 (20%)	<0.024

Medical history					

HTN	425 (32%)	491 (36%)	524 (46%)	669 (60%)	<0.0001
Diabetes	142 (11%)	120 (9%)	95 (8%)	155 (14%)	<0.0001
Current smokers	158 (12%)	165 (12%)	151 (13%)	157 (14%)	<0.31
Renal disease (self-report)	20 (1.5%)	17 (1.3%)	31 (2.7%)	45 (4.0%)	0.0001

Laboratory/clinical parameters					

Creatinine mg/dL	0.84 (0.2)	0.91 (0.2)	0.97 (0.2)	1.13 (0.5)	<0.0001
GFR MDRD mL/min/1.73 m^2^	91.8 (16.0)	84.0 (14.2)	78.4 (13.5)	67.8 (16.0)	<0.0001
Systolic BP, mm Hg	121.2 (20.2)	123.8 (20.0)	126.7 (21.0)	131.4 (22.9)	<0.0001
Diastolic BP, mm Hg	71.5 (10.5)	72.3 (10.2)	72.0 (9.9)	71.6 (10.6)	0.19
Body mass index Kg/m^2^	26.7 (4.8)	27.5 (4.7)	28.2 (4.9)	28.8 (5.2)	<0.0001
Body surface area m^2^	1.80 (0.2)	1.85 (0.2)	1.87 (0.2)	1.88 (0.2)	<0.0001

Medications					

Antihypertensive meds	322 (24%)	414 (31%)	421 (37%)	598 (54%)	<0.0001
Beta-blockers	70 (5%)	86 (6%)	110 (10%)	157 (14%)	<0.0001
ACE inhibitors	107 (8%)	113 (8%)	122 (11%)	199 (18%)	<0.0001
ARB	23 (2%)	41 (3%)	30 (3%)	67 (6%)	<0.0001
Calcium channel blockers	127 (9%)	146 (11%)	145 (13%)	174 (16%)	<0.0001
Thiazide diuretics	47 (3%)	70 (5%)	79 (7%)	126 (11%)	<0.0001
Loop diuretics	10 (1%)	11 (1%)	15 (1%)	40 (4%)	<0.0001

Continuous variables as mean (standard deviation) and categorical variables as percentages; *P* value obtained using analysis of variance and chi-square test across cystatin C quartiles. cystatin C quartiles 1: ≤0.76, quartile 2: >0.76 & ≤0.86, quartile 3: >0.86 & ≤0.98, quartile 4: >0.98 in mg/dL; HTN: hypertension; GFR: glomerular filtration rate; MDRD: modification of diet in renal disease; ACE: angiotensin-converting enzyme; ARB: angiotensin receptor blocker.

**Table 2:** The association of cystatin C with LV parameters in nested models showing *P* trend with increasing cystatin C quartiles.

Outcomes variables	Quartile 1	Quartile 2	Quartile 3	Quartile 4	*P* trend
Model 1 (*n* = 4,970)					

LVEDV, ML	125.7 ± 0.8	128.7 ± 0.9	127.0 ± 0.9	123.9 ± 1.0	0.12
LVESV, ML	39.5 ± 0.4	41 ± 0.5	40.2 ± 0.5	39.2 ± 0.5	0.55
LVM, Gm	138.0 ± 1.0	144.8 ± 1.1	148.2 ± 1.2	151.9 ± 1.2	0.0001
LVM/LVEDV, Gm/ML	1.11 ± 0.01	1.14 ± 0.01	1.19 ± 0.01	1.25 ± 0.01	0.0001
EF, %	69.2 ± 0.2	68.8 ± 0.2	69.0 ± 0.2	69.2 ± 0.2	0.90

Model 2 (*n* = 4,970)					

LVEDV, ML	128.1 ± 0.7	127.5 ± 0.7	125.5 ± 0.7	124.3 ± 0.8	0.0002
LVESV, ML	40.1 ± 0.4	40.1 ± 0.4	39.2 ± 0.4	39.2 ± 0.5	0.07
LVM, Gm	145.7 ± 0.8	145.1 ± 0.8	144.9 ± 0.8	148.0 ± 0.9	0.12
LVM/LVEDV, Gm/ML	1.15 ± 0.01	1.15 ± 0.01	1.17 ± 0.01	1.21 ± 0.01	0.0001
EF, %	69.4 ± 0.2	69.3 ± 0.2	69.4 ± 0.2	69.2 ± 0.2	0.71

Model 3 (*n* = 4,946)					

LVEDV, ML	128.2 ± 0.7	127.6 ± 0.7	125.5 ± 0.7	124.0 ± 0.8	0.0001
LVESV, ML	40.1 ± 0.4	40.1 ± 0.4	39.1 ± 0.4	39.0 ± 0.5	0.04
LVM, Gm	146.1 ± 0.8	145.7 ± 0.7	145.3 ± 0.8	147.3 ± 0.8	0.45
LVM/LVEDV, Gm/ML	1.15 ± 0.01	1.16 ± 0.01	1.17 ± 0.01	1.21 ± 0.01	0.0001
EF, %	69.5 ± 0.2	69.3 ± 0.2	69.5 ± 0.2	69.3 ± 0.2	0.75

Cystatin C quartiles 1: ≤0.76, quartile 2: >0.76 & ≤0.86, quartile 3: >0.86 & ≤0.98, quartile 4: >0.98 in mg/dL; LV parameters in adjusted least square mean ± standard error. LVEDV: LV end diastolic volume; LVESV: LV end systolic volume; LVM: LV mass; LVM/LVEDV: LV concentricity; EF: ejection fraction (fraction).

Model 1 univariate analysis; Model 2 adjusted for age, race/ethnicity, gender, height, and weight; Model 3 adjusted for Model 2 plus diabetes, use of antidiabetic medications, hypertension history, systolic blood pressure, total cholesterol, antilipid medications, and smoking.
